# Activation of PPARs *α*, *β*/*δ*, and *γ* Impairs TGF-*β*1-Induced Collagens' Production and Modulates the TIMP-1/MMPs Balance in Three-Dimensional Cultured Chondrocytes

**DOI:** 10.1155/2010/635912

**Published:** 2010-10-04

**Authors:** Paul-Emile Poleni, Stephanie Etienne, Emilie Velot, Patrick Netter, Arnaud Bianchi

**Affiliations:** ^1^Laboratoire de Physiologie et Pharmacologie Articulaires (LPPA), UMR 7561 CNRS-UHP Nancy 1, avenue de la Forêt de Haye, BP 184, 54505 Vandœuvre-lès-Nancy Cedex, France; ^2^Laboratoire de Biophotonique et Pharmacologie-UMR CNRS 7213, Faculté de Pharmacie, 74 route du Rhin, BP 60024, 67401 ILLKIRCH Cedex, France

## Abstract

*Background and Purpose*. We investigated the potency of Peroxisome Proliferators-Activated Receptors (PPARs) *α*, *β*/*δ*, and *γ* agonists to modulate Transforming Growth Factor-*β*1 (TGF-*β*1-) induced collagen production or changes in Tissue Inhibitor of Matrix Metalloproteinase- (TIMP-) 1/Matrix Metalloproteinase (MMP) balance in rat chondrocytes embedded in alginate beads. *Experimental Approach*. Collagen production was evaluated by quantitative Sirius red staining, while TIMP-1 protein levels and global MMP (-1, -2, -3, -7, and -9) or specific MMP-13 activities were measured by ELISA and fluorigenic assays in culture media, respectively. Levels of mRNA for type II collagen, TIMP-1, and MMP-3 & 13 were quantified by real-time PCR. *Key Results*. TGF-*β*1 increased collagen deposition and type II collagen mRNA levels, while inducing TIMP-1 mRNA and protein expression. In contrast, it decreased global MMP or specific MMP-13 activities, while decreasing MMP-3 or MMP-13 mRNA levels. PPAR agonists reduced most of the effects of TGF-*β*1 on changes in collagen metabolism and TIMP-1/MMP balance in rat in a PPAR-dependent manner, excepted for Wy14643 on MMP activities. *Conclusions and Implications*. PPAR agonists reduce TGF-*β*1-modulated ECM turnover and inhibit chondrocyte activities crucial for collagen biosynthesis, and display a different inhibitory profile depending on selectivity for PPAR isotypes.

## 1. Introduction

Articular cartilage is an avascular, aneuronal, and alymphatic connective tissue designed to distribute mechanical load, and provide a wear-resistant surface to the articular joints [1]. These biomechanical properties are supported by a specialized extracellular matrix which is maintained by chondrocytes, the unique cellular component of hyaline cartilage, under normal conditions of low turnover. Disruption of chondrocyte-matrix associations and alteration of metabolic responses in chondrocytes are thought to contribute to the pathogenesis of osteoarthritis [1], especially when the biosynthetic anabolic activity is unable to keep pace with the degradative catabolic activity [2]. Indeed, key pathological findings of osteoarthritic cartilage include excessive production of matrix degrading proteinases and proinflammatory cytokines, enhanced apoptosis, alteration of the chondrocytic phenotype, secondary osteophytes formation, decreased biosynthesis of cartilage specific molecules, and/or defective responses to growth factors [1]. The imbalance between chondrocyte functions is therefore thought to reflect the imbalance between catabolic cytokines and anabolic growth factors into diseased joints [3].

Transforming Growth Factor- (TGF-) *β* is a multifunctional growth factor [4]. In hyaline cartilage, TGF-*β* is produced in a latent form that does not activate TGF-*β* receptors but behaves as a great modulator of cartilage metabolism in its active form. Thus, it is a potent stimulator of chondrocyte proliferation [5] and differentiation [6] which stimulates cartilage-specific type II collagen or aggrecan synthesis [7–10] and modulates matrix metalloproteases activities by increasing synthesis of their natural tissue inhibitors [11, 12]. TGF-*β*1 may have a multifaceted physiopathological role in joint diseases depending variably on cartilage protection, bone remodelling and synovial expression suggesting the involvement of TGF-*β*1 in the evolution of osteoarthritis disease. 

Peroxisome Proliferators-Activated receptors (PPARs) are ligand-activated nuclear transcription factors belonging to the nuclear hormone receptor superfamily [13]. PPAR regulated genes involved in glucose and lipid homeostasis by binding to Peroxisome Proliferators-Response Elements (PPRE) located in their promoter. Three subtypes are encoded by separate genes, PPAR *α*, PPAR *β*/*δ*, and PPAR *γ* [14]. PPAR ligands differ greatly in their binding affinity between endogenous and synthetic agonists. Thus, arachidonic acid metabolites, such as leukotriene B4 for PPAR *α*, prostaglandin I_2_ for PPAR *β*/*δ*, or 15-deoxy-prostaglandin J_2_ (15d-PGJ_2_) for PPAR *γ* [15], are much less effective than pyrixin acid (Wy14643) or fibrates for PPAR *α*, GW501516 for PPAR *β*/*δ*, or thiazolidinediones for PPAR *γ* [16]. PPAR activation is associated with key functions including oxidation of fatty acids in liver (PPAR *α*) and skeletal muscle (PPAR *β*/*δ*) or differentiation of adipocytes in adipose tissue (PPAR *γ*), opening insight to the treatment of metabolic syndrome and cardiovascular diseases [17]. PPARs are also implied in the control of the inflammatory response [18] and epithelial repair pathways, with a foremost contribution of PPAR *β*/*δ* and PPAR *γ* to skin wound healing and liver or kidney fibrosis, respectively [19]. Their activation is associated with a reduced secretion of inflammatory cytokines and a lower activation of matrix metalloproteases [20] in articular cell culture or experimental arthropathy [21, 22]. PPAR agonists can also repress the synthesis of extracellular matrix components in quite a lot of cell types, such as collagen synthesis by thiazolidinediones in mesangial cells from diabetic rats [23] and Wy14643 in hepatic stellate cells from rats with cholestatic liver fibrosis [24]. Likewise, fibronectin expression is inhibited by pioglitazone in human kidney fibroblasts [25].

We investigated the ability of selective agonists of the three PPAR isotypes to affect TGF-*β*1-induced changes in collagen synthesis and TIMP-1/MMPs' balance in rat chondrocytes. In our culture system, the stimulatory effect of TGF-*β*1 on collagen deposition or type II collagen mRNA expression was dramatically reduced by all PPAR agonists. Wy14643 was solely associated with inhibition of basal MMP-3 & -13 mRNA expression and basal global MMP (-1, -2, -3, -7, and -9) or specific MMP-13 activities whereas all PPAR agonists reduced the stimulatory effect of TGF-*β*1 on TIMP-1. Our data demonstrates that PPAR agonists reduce TGF-*β*1-induced responses in chondrocytes and may favour an altered repair capacity of cartilage in response to growth factors. When considering the important role of TGF-*β*1 in cartilage repair, *in vivo* studies become obvious to assess whether the antianabolic effect of PPAR agonists could be counterbalanced by their potency to reduce synovitis and/or prevent osteophyte formation.

## 2. Materials and Methods

### 2.1. Chondrocyte Isolation and Culture in Alginate

Normal articular cartilage was obtained from femoral heads of Wistar male rats (150–175 g; Charles River, L'Arbresle, France) killed under dissociative anesthesia (ketamine (Mérial, Lyon, France) and acepromazine (Sanofi Santé Animale, Libourne, France)) in accordance with national animal care guidelines, after approval by our internal ethics committee. Chondrocytes were isolated by sequential enzymatic digestion of head caps using 0.15% pronase and 0.2% collagenase B (Roche Molecular Biochemicals, Meylan, France), then washed two times in phosphate buffered saline (PBS) and cultured up to confluence in 75 cm^2^ flasks at 37°C in a humidified atmosphere containing 5% CO_2_. The medium used was Dulbecco's Modified Eagle Medium (DMEM) (Invitrogen, Cergy Pontoise, France) supplemented with L-glutamine (2 mM), penicillin-streptomycin (50 *μ*g/mL), amphotericin B (0.5 *μ*g/mL), and 10% (v/v) heat-inactivated fetal calf serum (FCS) (Invitrogen, Cergy Pontoise, France). After two passages, chondrocytes were encapsulated in alginate beads according to Ballock et al. [26]. Briefly, the cells were suspended in a 1.2% sterile solution of medium viscosity alginate (Sigma-Aldrich, Saint Quentin Fallavier, France) at a concentration of 5.10^6^ cells per mL and dispensed drop-wise into a 102 mM CaCl_2_ solution *via* a 22-gauge needle attached to a syringe. After instantaneous polymerisation, beads were allowed to further their polymerization for 15 minutes in CaCl_2_ solution before three washes in sterile 0.15 M NaCl solution, followed by two washes in complete culture medium. The beads were maintained in complete culture medium for seven days at 37°C in a humidified atmosphere of 5% CO_2_ before experiments.

### 2.2. Study Design

After seven days of culture, beads were transferred into culture medium containing 1% (v/v) FCS and were maintained under low-FCS conditions throughout the experiments.

In a first set of experiments, the effect of selected concentrations of selective PPAR agonists was studied on TGF-*β*1-induced (10 ng/mL; Peprotech Tebu, Le Perrey en Yvelines, France) changes in collagen production and TIMP-1/MMPs balance. The selective PPAR agonists used were Wy14643 (100 *μ*M, Calbiochem, Meudon, France) as PPAR *α* agonists, GW501516 (100 nM, Alexis Biochemicals, Paris, France) as PPAR *β* */* *δ* agonists, and rosiglitazone (10 *μ*M, Cayman Chemical, Ann Arbor, MI) as PPAR *γ* agonists. Agonists were added 2 hours before TGF-*β*1 and biological responses were studied 48 hours (gene expression) or 72 hours (matrix metabolism) after addition of TGF-*β*1. PPAR ligands were dissolved in dimethyl sulfoxide (DMSO) (Sigma-Aldrich) and used at a final concentration of less than 0.1% in culture medium.

In a second set of experiments, control experiments were performed in the presence of specific antagonists (GW6471 (10 *μ*M; Tocris Bioscience, Paris, France) for PPAR *α* and GW9662 (10 *μ*M; Cayman Chemical) for PPAR *γ*) to evaluate the involvement of PPAR isotype in the anti-TGF-*β*1 potency of PPAR agonists on collagen deposition, type II collagen mRNA expression, and global MMP (-1, -2, -3, -7, and -9). PPAR ligands were dissolved in DMSO and used at a final concentration of less than 0.2% in culture medium.

### 2.3. DNA Measurement Assay

Cells were incubated for 24 h to 72 h at 37°C in the presence or absence of TGF-*β*1 and/or PPAR agonists (added 2 h before TGF-*β* *1* when coincubated) in low-FCS (1%) culture medium. After stimulation, alginate beads were dissolved in citrate-EDTA buffer (55 mM/50 mM; pH 7.6) for 10 min at 37°C, and then centrifuged at 10000 rpm for 15 seconds. The supernatants were discarded and the pellets were washed 5 times with PBS. Then the pellets were suspended in 100 *μ*L of Hoechst buffer (10 mM TRIS, 1 mM EDTA, and 0.1 M of NaCl, pH 7.4) before 5 series of freezing (nitrogen liquid)/defreezing (60°C, 5 min) for lysing cells. The samples were mixed with 2 ml of Hoechst solution (0.1 *μ*g/mL in final concentration, Hoechst 33528 FluoProbes, Interchim. France) and the measurement of absorbance at 456 nm on a fluorescence spectrophotometer (HITACHI F-2000, Science Tec., Courtaboeuf, Les Ulis, France) was used to calculate the amounts of total DNA in the samples using calf thymus DNA (0.05–0.5 *μ*g/mL) as a standard and cell-free alginate beads as a blank.

### 2.4. Regional Localization of Collagens in Beads

After stimulation with TGF-*β*1, in the presence or absence of PPAR agonists and/or PPAR antagonists, alginate beads were fixed for 4 h at 20°C in a 4% paraformaldehyde solution (pH 7.4) (Sigma-Aldrich) containing 100 mM sodium cacodylate and 10 mM CaCl_2_, then washed overnight in 100 mM sodium cacodylate buffer containing 50 mM BaCl_2_. Graded solutions of ethanol were used to dehydrate samples, then embedded in paraffin and cut into sections (5 *μ*m thickness), before staining with Sirius red (Direct Red 80, Sigma Aldrich) (0.1% w/v in picric acid) for the visualization of collagen synthesis. Sections (*n* = 5 beads per condition) were observed at a magnification 100x or 400x with a photonic microscope (Nikon type 104, Tokyo, Japan) and staining intensity was analyzed using NIH Image software (http://rsb.info.nih.gov/).

### 2.5. Assay for Matrix Metalloproteases Activities

After stimulation with TGF-*β*1, in the presence or absence of PPAR agonists, global MMP activities were measured in culture supernatants using fluorogenic substrates. Latent MMP were activated by adding 50 *μ*L of 4-aminophenylmercuric acetate (APMA, 1.5 mM in Tris-HCl (50 mM, pH 7.5) reaction buffer containing 1.5 mM NaCl and 5 mM CaCl_2_) to 50 *μ*L of culture supernatant. Then, 50 *μ*L of the fluorogenic MMP (-1, -2, -3, -7, and -9) substrate “DNP-Pro-Leu-Gly-Leu-Trp-Ala-D-Arg-NH_2_” (*λ*_excitation_ 280 nm, *λ*_emission_ 360 nm; Calbiochem, La Jolla, CA) or of the MMP-13 substrate “MCA-Pro-Cha-Gly-Nva-His-Ala-Dpa-NH_2_” (*λ*_excitation_ 325 nm, *λ*_emission_ 393 nm; Calbiochem, La Jolla, CA) were added to a final concentration of 30 *μ*M and 60 *μ*M, respectively. After 4 h of incubation in darkness at 37°C in a humidified atmosphere of 5% CO_2_, MMP-induced cleavage of substrates was measured with a Fluostar Optima spectrofluorimeter (BMG Labtechnologies, Champigny sur Marne, France) at emission wavelengths of 360 nm for total MMP and 393 nm for MMP-13.

### 2.6. Assay for Tissue Inhibitor of Metalloproteinase-1 (TIMP-1)

TIMP-1 levels were assessed in the same culture supernatants as for MMP activities using a commercially available rat enzyme-linked immunosorbent assay (Quantikine, R&D Systems, Minneapolis, MN) according to the manufacturer's instructions. This assay showed no significant cross-reactivity with other TIMPs and the limit of detection was 37.5 pg/mL.

### 2.7. RNA Extraction and Real-Time Polymerase Chain Reaction

Alginate beads were dissolved in citrate-EDTA buffer (55 mM/50 mM; pH 7.6) for 10 min at 37°C, and then centrifuged at 10000 rpm for 15 seconds. The supernatants were discarded and the pellets were washed 5 times with PBS before extraction of total RNA using RNeasy minikit (Qiagen, Valencia, CA). Total RNA (500 ng) were reverse transcribed in a final volume of 50 *μ*L containing 10 *μ*L of 5X First Strand Buffer, 20 *μ*M hexanucleotides random primers, 1 *μ*L of Recombinant Ribonuclease Inhibitor (RNA-se OUT 40 U/*μ*L), 2 *μ*L of dNTP (5 mM), and 1 *μ*L of Moloney Murine Leukemia Virus (M-MLV; 200 U/*μ*L) (Invitrogen) for 90 min at 37°C. To quantify type II collagen, MMP-3 & -13, and TIMP-1 mRNAs expression, real-time PCR was performed using the Lightcycler Technology (Roche Molecular Biochemicals) and the SYBRgreen master mix system (Qiagen, Courtaboeuf, France). The sequences of the primers used and corresponding product lengths are summarized in Table 1. The transcript level of the housekeeping gene RP29 was determined for each sample; data are expressed as the normalized ratio of mRNA level of each gene of interest over the RP29 gene.

### 2.8. Statistical Analysis

Data are expressed as mean ± standard error of the mean (SEM) of 3 to 10 values obtained from 1 to 3 separate experiments. Comparisons were made by ANOVA, followed by Fisher's protected least significant difference test using StatView 5.0 software (SAS Institute, Cary, NC). Statistically significant differences from the control are indicated as *P < .05, from TGF-*β*1-treated cells as ^#^ *P* < .05, and from “TGF-*β*1 + PPAR ligands”-treated cells as ^§^ *P* < .05.

## 3. Results

### 3.1. Effects of TGF-*β*1 and PPAR Agonists on Cell Viability and Proliferation

**Figure 1:**
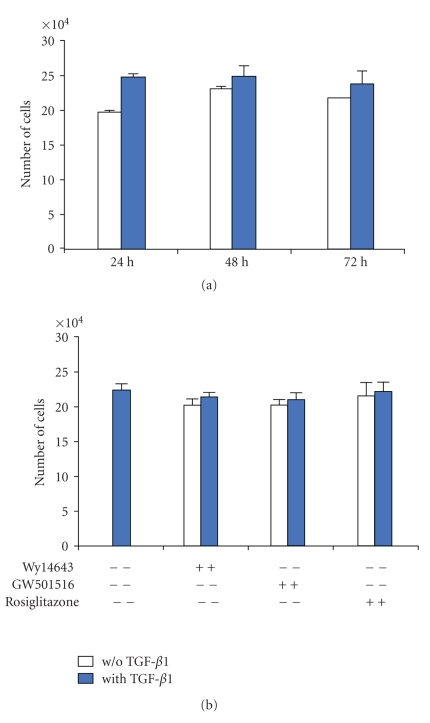
Effects of selective PPAR agonists on cell proliferation in rat chondrocytes stimulated with TGF-*β*1. Chondrocytes, embedded in alginate beads (5.10^4^ cells per bead), were exposed to Wy14643 (100 *μ*M), GW501516 (100 nM), or rosiglitazone (10 *μ*M) for 2 h before stimulation with TGF-*β*1 (10 ng/mL), in the presence or absence of PPAR agonists, for 72 h. Levels of total DNA were measured by Hoechst assay. (a) Time course effect of TGF-*β*1; (b) effect of PPAR agonists in presence of TGF-*β*1. Data are expressed as mean ± SEM.

In rat chondrocyte beads, TGF-*β*1 did not significantly increase cell proliferation as time went on (Figure 1(a)). We observed previously that Wy14643 behaved as a selective PPAR *α* agonist at 100 *μ*M, GW501516 as a selective PPAR *β* */* *δ* agonist at 100 nM, and rosiglitazone as a selective PPAR *γ* agonist [10]. In the same conditions of selectivity, PPAR agonists were found to be nontoxic for chondrocytes after 72 h of cell culture, in the presence or the absence of TGF-*β*1 (Figure 1(b)).

### 3.2. Effects of PPAR Agonists on TGF-*β*1-Induced Changes in Metabolism of Collagens

**Figure 2:**
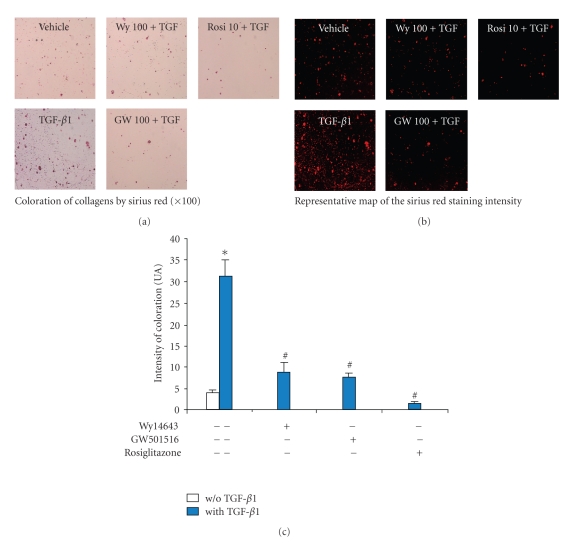
Effects of selective PPAR agonists on TGF-*β*1-induced collagens metabolism in rat chondrocytes. Chondrocytes, embedded in alginate beads (5.10^4^ cells per bead), were exposed to Wy14643 (100 *μ*M), GW501516 (100 nM), or rosiglitazone (10 *μ*M) for 2 h before stimulation with TGF-*β*1 (10 ng/mL), in the presence or absence of PPAR agonists for 72 h. (a) Representative sections of Sirius red staining of beads (*n* = 5, G: 100X); (b) Representative map of Sirius red staining intensity (*n* = 5); (c) Mean overall quantification of Sirius red staining on beads sections (*n* = 5). Data were expressed as mean ± SEM and statistically significant differences from the control were indicated as *P < .05 and from TGF-*β*1-treated cells as ^#^ *P* < .05.

**Figure 3:**
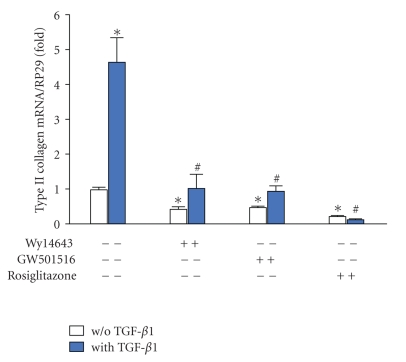
Effects of selective PPAR agonists on TGF-*β*1-induced type II collagen mRNA expression in rat chondrocytes. Chondrocytes, embedded in alginate beads (5.10^4^ cells per bead), were exposed to Wy14643 (100 *μ*M), GW501516 (100 nM), or rosiglitazone (10 *μ*M) for 2 h before stimulation with TGF-*β*1 (10 ng/mL), in the presence or absence of PPAR agonists for 48 h. Normalized mRNA levels of type II collagen were quantified by real-time PCR (*n* = 4). Data were expressed as mean ± SEM and statistically significant differences from the control were indicated as *P < .05 and from TGF-*β*1-treated cells as ^#^ *P* < .05.

TGF-*β*1 increased the intensity of Sirius red staining by 8.2-fold (Figures 2(a), 2(b), and 2(c)), while increasing the mRNA level of type II collagen by 4.5-fold (Figure 3). As expected from our experimental conditions, Sirius red staining was mainly localized around chondrocytes and highlighted the lack of any collagen fibers (Figures 2(a) and 2(b)).

PPAR agonists suppressed the basal staining for collagens or procollagens and reduced almost completely the TGF-*β*1-induced increase in staining intensity (Figures 2(b) and 2(c)). Accordingly, the stimulatory effect of TGF-*β*1 on type II collagen expression was completely suppressed by all agonists, with a maximal inhibitory potency for rosiglitazone (Figure 3). However, each agonist decreased the basal expression of type II collagen reaching an 80% inhibition for rosiglitazone (Figure 3).

### 3.3. Effects of PPARs Agonists on TGF-*β*1-Induced Changes in MMP/TIMP-1 Balance

**Figure 4:**
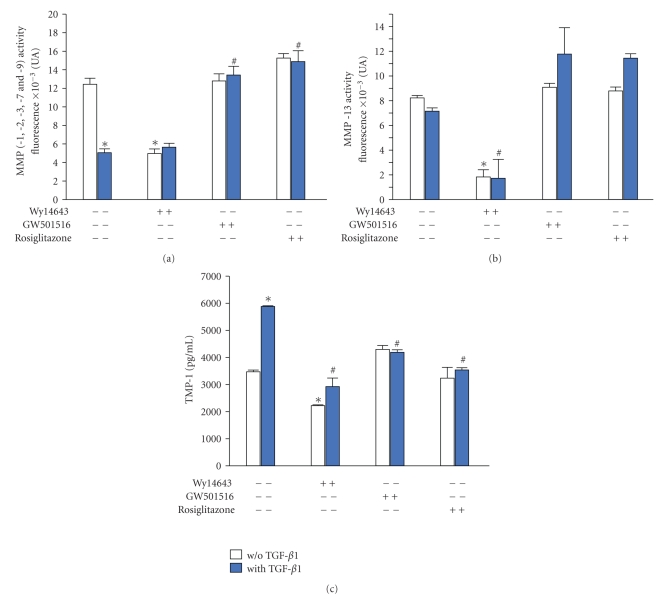
Effects of synthetic PPAR agonists on TIMP-1 production and changes of global MMPs (-1, -2, -3, -7, and -9) and specific MMP-13 activities induced by TGF-*β*1 in rat chondrocytes. Chondrocytes, embedded in alginate beads (5.10^4^ cells per bead), were exposed to Wy14643 (100 *μ*M), GW501516 (100 nM), or rosiglitazone (10 *μ*M) for 2 h before stimulation with TGF-*β*1 (10 ng/mL), in the presence or absence of PPAR agonists, for 72 h. (a) Global activity of MMPs (-1, -2, -3, -7, and -9) in culture supernatants by fluorimetric assay (*n* = 6); (b) Activity of MMP-13 in culture supernatants by specific fluorimetric assay (*n* = 6); (c) TIMP-1 protein levels in culture supernatants by ELISA (*n* = 3). Data were expressed as mean ± SEM and statistically significant differences from the control were indicated as *P < .05 and from TGF-*β*1-treated cells as ^#^ *P* < .05.

TGF-*β*1 decreased global MMP and MMP-13 activities by 66% and by 19%, respectively, (Figures 4(a) and 4(b)), while increasing TIMP-1 levels by 75% (Figure 4(c)). Consistently, the levels of mRNA for MMP-3 and -13 were reduced by 50% and by 10%, respectively, whereas those for TIMP-1 were upregulated by 65% (Table 2). 

In the presence of PPAR agonists, the inhibitory potency of TGF-*β*1 on MMP activities (Figures 4(a) and 4(b)) and mRNA levels (Table 2) was reversed completely by rosiglitazone and GW501516, while being unaffected by Wy14643. However, the basal MMP activities were decreased significantly by Wy14643 (Figures 4(a) and 4(b)), as well as the basal mRNA levels for MMP-3 and MMP-13 (Table 2). The stimulatory effect of TGF-*β*1 on TIMP-1 protein (Figure 4(c)) and mRNA (Table 2) levels was inhibited by all PPAR agonists with a major inhibitory potency for rosiglitazone and Wy14643. However, Wy14643 reduced the basal levels of TIMP-1 protein and mRNA by 40% and by 51%, respectively, (Figure 4(c); Table 2).

### 3.4. Reversion of the Antianabolic Potency of the PPAR Agonists by Selective Antagonists

Preliminary experiments using the MTT assay showed no loss of viability of rat chondrocytes exposed to PPAR agonists or PPAR antagonists, alone or in combination, in the concentration range tested (data not shown).

### 3.5. Effects on Collagen Metabolism

#### 3.5.1. Effects on Collagen Deposition

**Figure 5:**
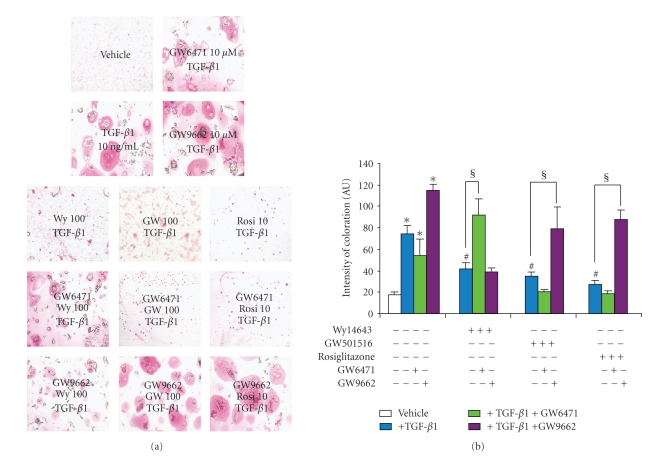
Selective reversion of anti-TGF-*β*1 potency of PPAR agonists on collagens' deposition in rat chondrocytes. Chondrocytes, embedded in alginate beads (5.10^4^ cells per bead), were pretreated for 2 h with Wy14643 (100 *μ*M), GW501516 (100 nM), or rosiglitazone (10 *μ*M), in the presence or absence of synthetic PPAR*α* (GW6471, 10 *μ*M) or synthetic PPAR*γ* (GW9662, 10 *μ*M) antagonists, before stimulation with 10 ng/mL TGF-*β*1 for 72 h. (a) Collagens' deposition in the extracellular matrix (cell-associated matrix) after coloration of representative sections of beads (*n* = 6, G: 400X) by Sirius red after 72 h. (b) Measurement of Sirius red staining of the representative section of beads using NIH/Image software (*n* = 4). Data were expressed as mean ± SEM and statistically significant differences from the control were indicated as *P < .05; from TGF-*β*1-treated cells as ^#^ *P* < .05; from “TGF-*β*1+agonists”-treated cells as ^§^ *P* < .05.

In our experimental condition (0.2% DMSO instead of 0.1%), the stimulatory effect of TGF-*β*1 (4.5-fold increase) on collagen deposition in the extracellular matrix (cell-associated matrix) was not affected by the PPAR*α* antagonist GW6471 but was potentiated by the PPAR*γ* antagonist GW9662 (6.8-fold increase) (Figures 5(a) and 5(b)). As observed previously, TGF-*β*1-induced collagens deposition was dramatically reduced by all PPAR agonists with a maximal inhibitory potency with rosiglitazone. In the presence of GW6471, the inhibitory potency of Wy14643 was completely relieved whereas those of GW501516 and rosiglitazone remained unchanged (Figures 5(a) and 5(b)). However, the inhibitory potency of rosiglitazone and GW501516 was totally suppressed by GW9662 whereas that of Wy14643 was not (Figures 5(a) and 5(b)).

#### 3.5.2. Effects on Type II Collagen mRNA Expression

**Figure 6:**
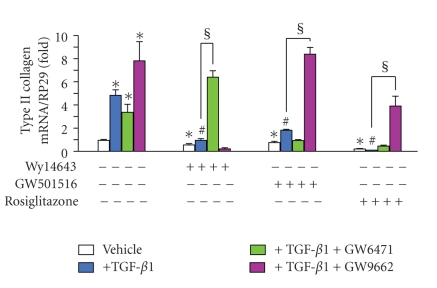
Selective reversion of anti-TGF-*β*1 potency of PPAR agonists on type II collagen mRNA expression in rat chondrocytes. Chondrocytes, embedded in alginate beads (5.10^4^ cells per bead), were pretreated for 2 h with Wy14643 (100 *μ*M), GW501516 (100 nM), or rosiglitazone (10 *μ*M), before stimulation with 10 ng/mL TGF-*β*1 for 48 h. Normalized collagen type II mRNA levels were quantified by real-time PCR (*n* = 4). Data were expressed as mean ± SEM and statistically significant differences from the control were indicated as *P < .05; from TGF-*β*1-treated cells as ^#^ *P* < .05; from “TGF-*β*1+agonists”-treated cells as ^§^ *P* < .05.

As for collagen deposition, the stimulatory effect of TGF-*β*1 on the mRNA level of type II collagen (5-fold increase) was not significantly affected by the PPAR*α* antagonist GW6471 but was potentiated by the PPAR*γ* antagonist GW9662 (8-fold increase) (Figure 6). Control experiments showed that GW6471 did not modify the basal level of type II collagen mRNA (data not shown), which was reduced by all PPAR agonists but markedly enhanced (2.4-fold) by GW9662 (Figure 6). In these conditions, TGF-*β*1-induced type II collagen expression was reduced by all PPAR agonists (from 75% for GW501516 to below basal level for rosiglitazone). In the presence of GW6471, the inhibitory potency of Wy14643 was relieved whereas that of GW501516 and rosiglitazone remained unaffected (Figure 6). However, the inhibitory potency of rosiglitazone and GW501516 was relieved in the presence of GW9662 whereas that of Wy14643 was accentuated.

#### 3.5.3. Effects on Global MMP (-1, -2, -3, -7, and -9)

**Figure 7:**
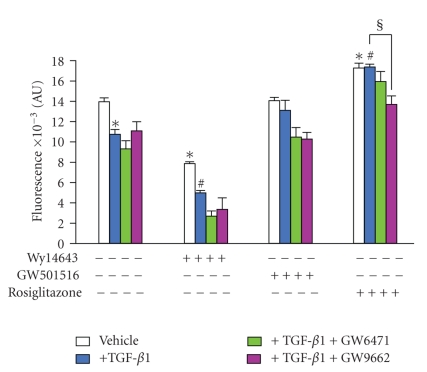
Selective reversion of anti-TGF-*β*1 potency of PPAR agonists on global MMP (-1, -2, -3, -7, and -9) activity in rat chondrocytes. Chondrocytes, embedded in alginate beads (5.10^4^ cells per bead), were pretreated for 2 h with Wy14643 (100 *μ*M), GW501516 (100 nM), or rosiglitazone (10 *μ*M), in the presence or absence of synthetic PPAR*α* (GW6471, 10 *μ*M) or synthetic PPAR*γ* (GW9662, 10 *μ*M) antagonists, before stimulation with 10 ng/mL TGF-*β*1 for 72 h. Global MMP (-1, -2, -3, -7, and -9) activities was evaluated by fluorigenic assays in culture supernatants (*n* = 4). Data were expressed as mean ± SEM and statistically significant differences from the control were indicated as *P < .05, from TGF-*β*1-treated cells as ^#^ *P* < .05, and from “TGF-*β*1+agonists”-treated cells as ^§^ *P* < .05.

As shown in Figure 7, TGF-*β*1-induced a loss of global MMP (-1, -2, -3, -7, and -9) activities which was reduced in our new experimental conditions (0.2% DMSO instead of 0.1%) but was significantly affected neither by GW9662 nor by GW6471. In these experimental conditions, the inhibitory effect of TGF-*β*1 on global MMP (-1, -2, -3, -7, and -9) activities was relieved by rosiglitazone, while being partly inhibited by GW501516 but moderately potentiated by Wy14643 (Figure 7). The anti-TGF-*β*1 potency of rosiglitazone was decreased selectively by GW9662. In contrast, that of GW501516 was relieved neither by GW6471 nor by GW9662, while that of Wy14643 was moderately accentuated (Figure 7). Taken all together, these data demonstrate the contribution of PPAR in the inhibitory potency of all PPAR agonists, excepted for Wy104643 which probably inhibited global MMP activities through PPAR-independent mechanism pathways.

## 4. Discussion

In the presence of PPAR agonists, the basal expression of type II collagen was decreased, in good accordance with their ability to prevent kidney [23, 27] or liver [24] fibrosis. More importantly, the stimulatory effect of TGF-*β*1 on its expression was reduced almost completely. Sirius red staining followed the same variation, suggesting that transcriptional inhibition by PPAR agonists resulted in decreased synthesis of collagen precursors. As for proteoglycans [10], this effect was seen independently of the expected selectivity for one PPAR isotype although maximal inhibition was observed for rosiglitazone. The contribution of PPAR*α* and PPAR*γ* was confirmed by the selective reversion of the inhibitory effect of Wy14643 by GW6471 and of rosiglitazone by GW9662, respectively. These data were consistent with the inhibitory effect of PPAR*γ* agonists on epithelial cells proliferation and ability to synthesize collagens in response to TGF-*β* [28–30]. 

Our data was more surprising for PPAR*α* agonists since Wy14643 was unable to suppress the inducing effect of TGF-*β*1 on collagen type I in pulmonary myofibroblasts [30]. As these data were obtained from different cell types (myofibroblasts *versus* chondrocytes) and for different collagens (type I *versus* type II), it might be suggested that this discrepancy could reflect cell specificities and/or result from a different regulation of collagen genes by TGF-*β*. However, a regulatory role of PPAR *α* on the synthesis of extracellular matrix components is supported indirectly by the enhanced matrix formation found in the kidney of PPAR *α*-deficient mice developing diabetes [31]. At the concentration used, Wy14643 was thought to inhibit collagen synthesis by a PPAR *α*- and *γ*-dependent mechanism in hepatic stellate cells [24], but such dual inhibitory effect was not supported by our reversion study with the PPAR *α* antagonist GW6471. Our data were less clear for PPAR*β*/*δ* since the inhibitory effect of GW501516 on TGF-*β*1-induced expression of type II collagen was reversed by the PPAR *γ* antagonist GW9662. It could not be excluded that GW9662 behaved as a moderate PPAR *β*/*δ* antagonist in our culture system [32]. Data are lacking on the contribution of PPAR *β*/*δ* to the synthesis of extracellular matrix components, but its expression was found to be regulated antagonistically by TGF-*β*1 and inflammatory cytokines during wound healing [33], suggesting that it could interfere with matrix turnover as a result of signalling cross-talks. Taken together, our data demonstrated that activation of any PPAR isotype reduced the stimulatory effect of TGF-*β*1 on collagen synthesis, in the context of a broader suppressive effect on the synthesis of cartilage specific components. As type II collagen concentration was shown to mediate part of the stimulatory effect of TGF-*β*1 on its own expression [34], PPAR agonists might reduce extracellular matrix synthesis through direct and indirect mechanisms in chondrocytes.

The presence of multiple TIMPs including TIMP-1 [35] or TIMP-3 [36] in normal human synovium and TIMP-2 in adult cartilage [35] may protect the integrity of the extracellular matrix. The *in vivo* increase in TIMP-3 and TIMP-1 messengers may be mediated by factors in joints such as serum, IL-1*β*, and TGF-*β*1, which also induce them *in vitro* [37, 38]. TIMP-1 and TIMP-3 may be beneficial for normal joints; however, their increase in human or bovine arthritic joint has been associated with pathological remodelling [36]. 

Concomitantly to the increase of TIMP-1, TGF-*β*1 has been shown to inhibit several MMP expression or activity. Indeed, TGF-*β*1 decreased MMP-1 and MMP-3 in human normal cartilage [39]. The clinical manifestations of an imbalance between TIMPs and tissue degrading enzymes are evident in a variety of pathological states, in which inadequate cartilage remodelling is obvious [40, 41]. In our report, we found that MMP-3 mRNA expression and MMP (-1, -2, -3, -7, and -9) activities were decreased, while a slight downregulation of MMP-13 mRNA expression or activity was observed. Taken together, TGF-*β*1 appears to be an anticatabolic factor in normal rat chondrocytes cultured in alginate beads by decreasing MMP expressions, suggesting the important role of this growth factor in extracellular matrix- (ECM-) cartilage synthesis and turnover. Our data showed that PPAR ligands differentially regulated MMP expression or activities in presence or absence of TGF-*β*1. In the presence of PPAR agonists, the inhibitory potency of TGF-*β*1 on MMP activities and mRNA levels was shown to be completely reversed by rosiglitazone and GW501516, while being unaffected by Wy14643. Nevertheless, in our experimental conditions, the basal MMP activities were decreased significantly by Wy14643, as well as the basal mRNA levels for MMP-3 and MMP-13, demonstrating that the regulation of expression or activities of MMP were dependent on the selectivity profile of each PPAR agonist. Taken together, only Wy14643 appeared to protect cartilage directly from collagens degradation by reducing MMP' expression or activities.

Our data demonstrate for the first time that PPAR agonists reduce TGF-*β*1-induced changes in collagen metabolism and TIMP-1/MMPs balance. These agonists modulate ECM turnover and inhibit chondrocyte activities crucial for collagen biosynthesis, and display a different inhibitory profile depending on selectivity for PPAR isotypes, especially for MMP. Our data might suggest that PPAR agonists as Wy14643 might be beneficial for preventing the progression of joint pathologies by protecting extracellular matrix components from catabolic factors. PPAR agonists showed anti-inflammatory and anticatabolic properties in animal models of adjuvant-induced arthritis where PPAR agonists reduced synovial inflammation, while preventing cartilage destruction or loss in bone mineral density [42]. A recent *in vitro *study demonstrated an inhibition of the expression of PPAR *γ* in human osteoarthritic cartilage and its downregulation by IL-1*β* *via* a mechanism involving activation of MAPKs (p38 and JNK) and NF*κ*B signalling pathways, suggesting that upregulation of PPAR *γ* might be beneficial in the context of preventing osteoarthritis [43]. Physiological findings of inflammatory experimental models of osteoarthritis demonstrated that 30 mg/day pioglitazone, a PPAR *α* > *γ* agonist [10, 42], ameliorated most aspects of joint lesions of the femoral condyles rather than those of the tibial plateaus [22, 44]. Although the exact reason of the difference in effectiveness of pioglitazone remain unclear, the effect of this agonist was correlated with a reduction of inflammation while preventing cartilage erosion *via* inhibition of synthesis or activity of catabolic factors, such as MMP (-1 & -13) or ADAMTS-5 [44]. In the present work, we demonstrated that PPAR agonists (Wy14643) are capable to inhibit mRNA expression or activity of MMP-3 & 13 and global MMP activity through a PPAR-independent mechanism pathway. Although upregulation of PPAR *γ* has been reported to be beneficial in the context of preventing osteoarthritis and arthritis, the PPAR-independent mechanism contribution to the anticatabolic potency of the PPAR agonists may be in good agreement with the absence of any relationship between the polymorphism PPAR*γ* or the severity of osteoarthritis [45]. Furthermore, since clinical data reported that rheumatoid arthritis patients treated with PPAR agonists for type 2 diabetes or dyslipidemia are paradoxically suffering from osteoarthritis [46], the proof of concept that PPAR agonists have therapeutical relevance may benefit from an epidemiological study of joint lesions in patients with advanced osteoarthritis treated for long periods of time with PPAR *α* > *γ* (pioglitazone) or PPAR *α* (fibrate, Wy14643) agonists. In that case, we hope that the present work could be taken into consideration in the establishment of new targeted therapies or protocols dedicated to the treatment of advanced arthritic and/or osteoarthritic patients with less safety concern.

## Figures and Tables

**Table 1:** Primers used for real-time PCR and corresponding product lengths.

Target genes	Primer sequence Forward	Primer sequence Reverse	Tm (°C)	Length (Pb)
RP 29	5^**'**^-AAG ATG GGT CAC CAG CAG CTC TAC TG-3^**'**^	5^**'**^-AGA CGC GGC AAG AGC GAG AA-3^**'**^	59	67
Type II Collagen	5^**'**^-TCC CTC TGG TTC TGA TGG TC-3^**'**^	5'-CTC TGT CTC CAG ATG CAC CA-3^**'**^	59	161
TIMP-1	5^**'**^-TCC CCA GAA ATC ATC GAG AC-3^**'**^	5^**'**^-TCA GAT TAT GCC AGG GAA CC-3^**'**^	61	159
MMP-3	5^**'**^-GCT CAT CCT ACC CAT TGC AT-3^**'**^	5^**'**^-GCT TGT GCA TCA GCT CCA TA-3^**'**^	58	219
MMP-13	5^**'**^-AGG CCT TCA GAA AAG CCT TC-3^**'**^	5^**'**^-GAG CTG CTT GTC CAG GTT TC-3^**'**^	58	226

**Table 2:** Effects of selective PPAR agonists on TGF-*β*1-induced changes in MMPs and TIMP-1 genes expression in rat chondrocytes. Chondrocytes, embedded in alginate beads (5.10^4^ cells per bead), were exposed to Wy14643 (100 *μ*M), GW501516 (100 nM), or rosiglitazone (10 *μ*M) for 2 h before stimulation with TGF-*β*1 (10 ng/mL), in the presence or absence of PPAR agonists, for 48 h. Normalized mRNA levels of MMP-3, MMP-13, and TIMP-1 quantified by real-time PCR (*n* = 4). Data are expressed as mean ± SEM. Statistically significant differences from the control are indicated as *P < .05, and from TGF-*β*1-treated cells as ^#^ *P* < .05.

PPAR agonists		Genes/RP29	
	MMP-3	MMP-13	TIMP-1
None			
w/o TGF*β*l	1.00 ± 0.04	1.00 ± 0.03	1.00 ± 0.06
+ TGF*β*l	0.45 ± 0.04*	0.89 ± 0.02	1.65 ± 0.07*
Wy14643			
w/o TGF*β*l	0.54 ± 0.01*	0.13 ± 0.03*	0.49 ± 0.04*
+ TGF*β*l	0.56 ± 0.06	0.44 ± 0.10^#^	0.71 ± 0.21^#^
GW501516			
w/o TGF*β*l	1.28 ± 0.08	1.52 ± 0.12*	1.10 ± 0.14
+ TGF*β*l	1.45 ± 0.07^#^	1.68 ± 0.05^#^	1.38 ± 0.05
Rosiglitazone			
w/o TGF*β*l	1.24 ± 0.20	1.24 ± 0.02	0.76 ± 0.03*
+ TGF*β*l	1.40 ± 0.18^#^	1.44 ± 0.03^#^	1.00 ± 0.14^#^
